# Inspiratory versus expiratory incentive spirometry: A randomised control trial study protocol

**DOI:** 10.4102/sajp.v79i1.1841

**Published:** 2023-10-09

**Authors:** Eniola O. Awolola, Sonil S. Maharaj

**Affiliations:** 1 Department of Physiotherapy, Lagos State University Teaching Hospital, Lagos, Nigeria; 2 Department of Physiotherapy, College of Health Sciences, University of KwaZulu-Natal, Durban, South Africa

**Keywords:** respiratory impairment, incentive spirometry, pulmonary function test, six-minute walk test (6MWT)

## Abstract

**Background:**

Respiratory impairments refer to a reduction in pulmonary function, which may adversely affect an individual’s health. Incentive spirometry is a technique designed to assist patients in achieving a pre-set airflow volume; the volume is determined from predicted values or baseline measurements. Our study aims to assess the effect of incentive spirometry on respiratory impairments.

**Method:**

Fifty-four patients aged 40 years and above with obstructive, restrictive or mixed respiratory impairments attending the respiratory clinic at the Lagos State University Teaching Hospital, Ikeja (LASUTH), will be recruited and assigned to three groups of 18 participants based on the class of respiratory impairment. Participants in each category of respiratory impairment will be subdivided into three groups. A final group of six participants per class of impairment will participate in the experiment. Our study will be a double-blind, randomised control trial with two intervention groups and one parallel placebo control group. Pulmonary function will be assessed before and after every procedure while the six-minute walk test (6MWT), Medical Research Council dyspnoea scale and the Pulmonary Functional Status and Dyspnea Questionnaire-Modified will be assessed fortnightly during our study. Data will be analysed using descriptive and inferential statistics and a repeated MANOVA; *p* < 0.05.

**Discussion:**

The outcome of our study may reveal the effect of inspiratory and expiratory incentive spirometry on obstructive, restrictive or mixed respiratory impairments.

**Conclusion:**

Our study may contribute to the body of knowledge on pulmonary rehabilitation.

**Clinical implication:**

Our study results may indicate if inspiratory incentive spirometry or expiratory incentive spirometry is better suited for the treatment of the respiratory impairment.

**Trial Registration:**

www.pactr.org: PACTR202005904039357

## Background

Respiratory impairment is a common condition seen by healthcare service providers in primary healthcare, pulmonary medicine and other specialties. It is more common in older persons and the aging population (Vaz Fragoso & Gill 2012).

Respiratory diseases may lead to a significant reduction in the timed lung volume; the forced expiratory volume in 1 sec to forced vital capacity ratio (FEV_1_/FVC) is reduced and defines airflow limitations because of airway obstruction (Pellegrino et al. 2005). Respiratory diseases that lead to comparable reductions in the timed and untimed lung volumes include those that affect the chest wall and respiratory muscles, leading to a normal FEV_1_/FVC but reduced FVC (Pellegrino et al. 2005).

According to Fletcher (2019), respiratory symptoms such as dyspnoea have been found in a quarter to a third of the adult population with respiratory conditions such as chronic obstructive pulmonary disease (COPD). Chronic obstructive pulmonary disease is a group of progressive lung diseases commonly associated with emphysema, chronic bronchitis or both and are also associated with adverse outcomes such as increased disability and increased risk of death in the aging population (Agusti 2005; Soriano et al. 2005). Emphysema can lead to airway obstruction following the destruction of air sacs, while mucus build-up from inflammation can occur in bronchitis (Agusti 2005; Soriano et al. 2005).

Cystic fibrosis and idiopathic pulmonary fibrosis (IPF) are classified as restrictive airway diseases characterised by scarring of the lung tissues leading to a progressive decline in lung function (Liao et al. 2017). Interstitial lung disease (ILD) manifests as inflammatory, fibrotic infiltration of the alveolar walls resulting in the loss of lung tissue wall compliance (Nwosu et al. 2020). According to Anyabolu et al. (2013), the prevalence of IPF in the United States is 14 per 100 000; however, a scarcity of data is seen in the Nigerian population. A retrospective study between January 2015 and October 2018 by Nwosu et al. (2020) revealed that ILD accounted for 2.3% of respiratory conditions.

Diagnosis of respiratory diseases is frequently established through spirometry because pathological confirmation is invasive and not routinely available. Hence, airflow obstruction or restrictive patterns collectively referred to as respiratory impairment is often detected through spirometry (Marcus et al. 2015). In summary, respiratory conditions such as asthma and COPD are classified as obstructive diseases, while those involving the chest wall, respiratory muscles, pleura or lung parenchyma are considered restrictive in nature (Vaz Fragoso & Gill 2012).

The diagnostic criteria that define spirometry respiratory impairment are often based on the Global Initiative for Obstructive Lung Disease (GOLD) (Quanjer et al. 1993). The threshold of < 0.70 for the spirometry ratio of FEV_1_ to FVC classifies normal spirometry, airflow obstruction or restriction in the adult population (Leivseth et al. 2013, Quanjer et al. 1993, Vaz Fragoso & Gill 2012). Airway obstruction is defined with an FEV_1_/FVC ratio of < 70% and an FVC of > 80% predicted, FEV_1_/FVC ratio of > 70% and an FVC of < 80% predicted for restrictive defects, while mixed defects are identified with FVC of < 80% predicted and an FEV_1_/FVC ratio of < 70% (Mathew et al. 2016; Uzma et al. 2008).

Based on the controversies surrounding the utilisation of Inspiratory Incentive Spirometry (IIS) and Expiratory Incentive Spirometry (EIS) in the management of respiratory impairments, our study was conceived to compare the effect of inspiratory and expiratory incentive spirometry exercise on the pulmonary parameters and quality of life (QoL) of patients with obstructive, restrictive or mixed respiratory impairments and to determine the most suitable intervention for the respective respiratory impairments.

According to Parshall et al. (2012), respiratory impairment is typically established by conducting a pulmonary function test (PFT) using a spirometer and subsequently categorised as airflow limitation (e.g. COPD or asthma) or airflow restriction (e.g. interstitial lung disease or heart failure). The criteria that define this airflow limitation are based on the GOLD (Rabe et al. 2007).

Based on American Thoracic and European Respiratory Societies (ATS/ERS) performance guidelines for pulmonary function tests, a portable handheld device can be utilised for spirometry by instructing the participant to perform a series of forceful and complete exhalation manoeuvres, starting from maximal inspiration with the breathing manoeuvres generating two specific lung volumes, namely the FVC (an untimed lung volume) and FEV_1_ (a timed lung volume) (Pellegrino et al. 2005).

In managing respiratory diseases, a lack of awareness, knowledge and often delay in recognition of the disease is one of the reasons why primary care practitioners and other healthcare providers may incorrectly diagnose or manage the condition (Yawn & Wollan 2008). The pulmonary function test is a simple and accurate tool to assess airflow obstruction. Patients FEV_1_/FVC ratio is reduced, and FEV_1_ is reduced in COPD (Gold & Koth 2015). An airway reversibility test differentiates COPD from asthma, as COPD patients do not show reversibility in airflow obstruction after administration of bronchodilators (Vestbo et al. 2013).

## Significance of our study

The outcome of our study may establish the relationship between inspiratory and expiratory types of incentive spirometry in the management of obstructive, restrictive and mixed respiratory impairments.

Although studies have identified the general effect of incentive spirometry (IIS and EIS) in the management of respiratory conditions, none has been able to exclusively compare the impact of IIS with EIS on the different classes of respiratory impairments, thereby limiting the opportunity to identify the most suitable treatment modality for respiratory impairments. Our study is therefore designed to answer the following question.

What will be the effect of inspiratory and expiratory incentive spirometry on pulmonary function test (PFT), six-minute walk test (6MWT), Medical Research Council (MRC) dyspnoea scale and the Pulmonary Function Status and Disability Questionnaire-modified (PFSDQ-M) on patients with obstructive, restrictive or mixed respiratory impairments?

Thus, the aim of our study is to determine the effect of inspiratory and expiratory type of incentive spirometry on PFT, 6MWT, MRC dyspnoea scale score and PFSDQ-M score of patients with obstructive, restrictive or mixed respiratory impairments.

The specific objectives of our study are to determine:

- The effect of inspiratory incentive spirometry on FEV_1_, FVC, FEV_1_/FVC, PEFR, 6MWT, MRC dyspnoea scale score and PFSDQ-M score of patients with obstructive, restrictive or mixed respiratory impairments.
- The effect of expiratory incentive spirometry on FEV_1_, FVC, FEV1/FVC, PEFR, 6MWT, MRC dyspnoea scale score and PFSDQ-M score of patients with obstructive, restrictive or mixed respiratory impairments.

## Method

### Study Design

Our study will be a parallel 12-weeks randomised control trial, involving three groups; two intervention groups (IIS and EIS) and a parallel placebo control group of participants attending the respiratory clinic at the Lagos State University Teaching Hospital, Ikeja (LASUTH).

### Participants

The participants will consist of male and female patients aged 40 years and above attending the respiratory clinic of Lagos State University Teaching Hospital, Ikeja, Lagos, Nigeria, West Africa, with spirometry confirmed obstructive, restrictive or mixed respiratory impairment for a period greater than 6 months.

The inclusion criteria are patients with COPD, IPF, ILD, cystic fibrosis, thoracic cage abnormalities (kyphosis, kyphoscoliosis, pectus carinatum or pectus excavatum) history of about or more than 6 months duration attending the respiratory clinic of LASUTH, Ikeja, Lagos. The respiratory condition of the selected participants must be spirometry confirmed to have resulted in an obstructive, restrictive or mixed respiratory airway impairment.

The exclusion criteria are patients who are on a cardiac pacemaker, supplemental oxygen therapy, those with cardiac conditions, patients with psychological impairments and patients with bronchial asthma.

### Sample size calculation

**FIGURE 1:**
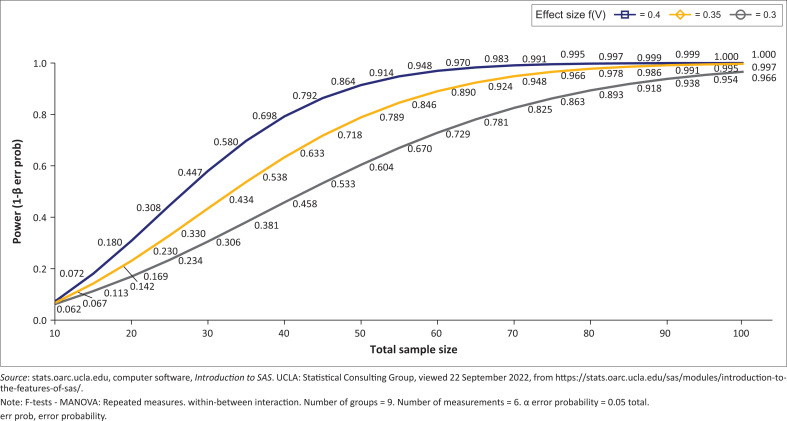
F-test multivariate analysis of variance of within/between group interaction.

The pulmonary function test is the primary outcome of interest for our study and the expected clinically relevant difference for obstructive restrictive and mixed impairment using LLN and GLI reference equation as proposed by Quanjer et al. (1993). Therefore, the sample size (*n*) will be determined using G-Power statistics software (stats.oarc.ucla.edu). The power is selected at 95% = 0.95, confidence level at 5% = 0.05 and effect size of 0.35 (Figure 1), namely:

**F-tests – MANOVA:** Repeated measures, within-between interaction

**Options:** Pillai V, O’Brien-Shieh Algorithm

**Analysis:** A priori: Compute required sample size

**Input:** Effect size f(V) = 0.35

    α err prob = 0.05

    Power (1-β err prob) = 0.8

    Number of groups = 9

    Number of measurements = 6

**Output:** Non-centrality parameter λ = 31.2375000

     Critical F = 1.4522092

     Numerator degree of freedom (*df*) = 40.0000000

     Denominator df = 210

     Total sample size = 51

     Actual power = 0.8010637

     Pillai V = 0.5456570

We intend to investigate the main effects, within and between interactions, for two factors, namely:

- Factor A – Therapy with three levels (Therapy A, Therapy B and No Therapy)
- Factor B – Types of impairments (Obstructive, Restrictive and Mixed) nested within factor A

This gives 3 × 3 = 9 groups of participants from which repeated measurements are to be obtained.

We are also planning to measure three continuous outcomes at different time points of which the minimum repeated measurements are expected to be six per subject for some of the outcomes and a maximum of 36 per subject. Suppose a minimum of six repeated measures is to be obtained from each subject, in that case, it is estimated that a minimum total sample size of 51 patients is required to detect an effect size v = 0.35 with 95% confidence (5% Type I) – similar studies also expected to produce similar results about 80% of the time (Power of test 80%). In the case that during the time of data collection, the hospital experiences a high number of COPD, IPF, ILD, cystic fibrosis, thoracic cage abnormalities patients or resources permit, we are able to increase the total sample size to at most 68 subjects with a corresponding increase in the power of the test to about 95% still detecting effect sizes between 0.3 and 0.4 (see Figure 1). This translates to the possibility of recruiting between five and eight patients per group. Hence, the sample size estimates suggest that recruiting more than eight patients per group for our study will be a waste of resources.

### Procedure

#### Randomisation and blinding

The patients attending the respiratory clinic of LASUTH, Ikeja, Lagos, Nigeria, West Africa, will be approached for possible interest in participating in our study. A bulk text message captioned ‘Invitation to a study on respiratory impairments’ will be circulated to interested participants using a Luxury bulk SMS platform.

**FIGURE 2:**
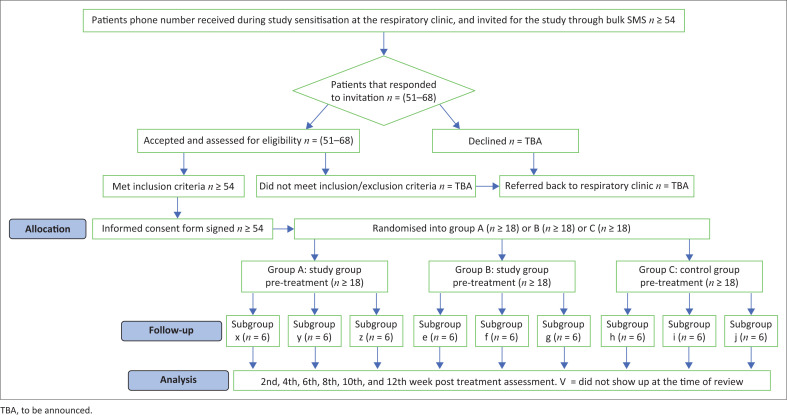
Consort flow diagram for recruitment and randomisation of participants.

Interested potential participants will be screened by Examiner 1 (the study physician) using the inclusion and exclusion criteria to determine their suitability. If suitable, they will be assigned to three groups of 18 participants per group based on the class of respiratory impairment by research assistant 1 (RA1). A simple random sampling technique will be used to assign interested participants into three major groups (groups A, B and C): subgroup A (x, y, z), subgroup B (e, f, g) and subgroup C (h, i, j). The allocation will be performed in phases through balloting according to the class of respiratory impairment, with each participant picking a slip of paper in a ballot box containing equal pieces marked either ‘A’ ‘B’ or ‘C’ for the main group, ‘x’ ‘y’ or ‘z’ for subgroup A, ‘e’ ‘f’ or ‘g’ for subgroup B and ‘h’ ‘i’ or ‘j’ for subgroup C, based on the number of participants present (Figure 2). The first author, who is the principal investigator (PI) will generate the ballot slip, while research assistant 2 (RA2) will supervise the balloting. There will be pre-commencement training for the field workers involved in the randomisation, investigation and data collection.

## Description of equipment, procedure and outcome measures

### Inspiratory incentive spirometry

Evidence from a Medline search from 1952 to 2008, EMBASE search from 1980 to 2008 and CINAHL search from 1980 to 2008 using OVID interface and Cochrane library search revealed several articles with incentive spirometry as a reliable tool for lung expansion in the postoperative physiotherapy rehabilitation of chest conditions (Agostini & Singh 2009).

Different types of incentive spirometer are available in the market; the volume-oriented incentive spirometer designed to improve lung volume has a visible scale that monitors the inspiratory effort of the patients (Agostini & Singh 2009). Mediflo Duo (Mediflex, Hamburg, Germany), Mediciser (Eastern Medikit Ltd., Gurgaon, India), Coach 2 Device (MediMark Europe, Grenoble, France), Spiroball (Leventon, Barcelonia, Spain) and several others are brands of incentive spirometry for inspiratory, volume-oriented respiratory exercises (Agostini & Singh 2009).

Following successful allocation to one of the three groups, the participants will be required to breathe in the upright sitting position before commencing the exercise using the most convenient respiration method. A volume-incentive spirometer (Coach 2 device), which enables the patient to inhale air through a mouthpiece and corrugated tubing attached to plastic bellows, will be used for the inspiratory exercise, with the volume of air displaced indicated on a scale located on the device enclosure (Kumar et al. 2016). The procedure will be demonstrated to the participant to ensure that it is well understood before commencement. After the participants have achieved the maximum volume, they will be instructed to hold the volume of air attained on the scale constant for 3–5 sec before exhalation (Zayed, Ahmed & Salem 2017). The participants will repeat the exercise three times to complete one set; three sets will be performed up to a maximum of 10 attempts. One minute of rest will be given between sets to reduce fatigue. The participants will leave after the following musculoskeletal assessment: The musculoskeletal assessment will involve screening of the participants’ musculoskeletal system for possible pain, swelling or limitation in joint range of motion. The procedure will be stopped whenever the subject feels dizziness or experience pain in the chest. The procedure will be repeated three times a week for 12 weeks. The MRC dyspnoea scale, 6MWT and PFSDQ-M will be conducted fortnightly during the study period.

### Expiratory incentive spirometry

Peak flow meters are portable hand-held device designed to measure how fast an individual can blow out air from the lungs during forceful exhalation, the peak flow meter evaluates airflow through the airways and monitors the degree of airway obstruction (Adeniy & Saminu 2011). The low range peak flow meter and the standard range peak flow meter are the two major types of peak flow meters; the low range is specific for children between 4 and 9 years and adults with severely impaired lung function, while the standard type of peak flow meter is used for older children, teenagers and adults (Adeniyi & Saminu 2011)

In a double-blind study by Nazir et al. (2005), on the accuracy of peak flow meters, a strong positive correlation was found in the comparison of three peak flow meters A, B and C with Pearson’s product moment correlation coefficient (r) = 0.962 for product A versus B, (r) = 0.969 for B versus C and (r) = 0.961 for A versus C. However, the disagreement between the Bland and Altman plot indicates that the peak flow meter should not be interchanged in clinical practice in order to maintain its accuracy and reliability.

Following successful allocation, the participants will be required to breathe in the upright sitting position before commencing the exercise using the most convenient respiration method. In an upright sitting position, the participants will be asked to breathe in air through the nose to the maximum, then breathe out into a standard peak flow meter. The peak flow cursor will be adjusted to zero, and participants will be instructed to avoid touching the cursor during the exercise. The procedure will be demonstrated to the participants to ensure that it is well understood before commencement. They will be required to wrap their mouth tightly around the mouthpiece of the peak flow meter, to prevent the slippage of air around the corners of the device, then breathe out maximally into the peak flow meter as hard and as fast as possible (Adeniyi & Erhabor 2011). The effort will be noticed on the cursor. The cursor will be returned to zero, and the exercise will be repeated three times to complete one set. A total of three sets will be performed up to a maximum of 10 attempts. One minute of rest will be given between sets to reduce muscular fatigue. The procedure will be stopped if the participant feels dizziness or experiences pain in the chest. To avoid a Valsalva manoeuvre, participants will be asked not to hold their breath for more than 5 seconds following the maximum expiration. The participant will leave after the following musculoskeletal assessment. The musculoskeletal assessment will involve screening of the participants’ musculoskeletal system for possible pain, swelling or limitation in joint range of motion. The procedure will be repeated three times a week for 12 weeks. The participants MRC dyspnoea scale, 6MWT and PFSDQ-M will be conducted fortnightly during the study period.

### Control group

Placebo effect refers to treatment that is essentially not therapeutically effective (Rossettini et al. 2020).

Following successful allocation, participants will be required to breathe in the upright sitting position using a respiration method that is most convenient for them. An assessment of the participants’ musculoskeletal system will be conducted as a placebo immediately after their pulmonary function test is conducted. The musculoskeletal assessment will involve a medical screening of the participants’ musculoskeletal system for possible pain, swelling or limitation in joint range of motion. The participant will leave after the musculoskeletal examination. The procedure will be repeated three times a week for 12 weeks. The MRC dyspnoea scale, 6MWT and PFSDQ-M will be conducted fortnightly during our study period.

### Spirometry

The assessment will be conducted by a technician certified by the Pan African Thoracic Society, blinded to group allocation. A portable spirometer (Koko SX 1000 Standalone Version 7 Pneumotach) will be used for this assessment. Daily calibration of the device will be conducted using a 3.0-L syringe. A brief description of the assessment procedure and technical steps to obtain pulmonary function data and variables will be explained to each participant. After 2–3 tidal breaths, participants will be asked to inhale deeply to total lung capacity and then exhale rapidly (without any pause) through a disposable mouthpiece until as much air as possible has been expelled. The test will be performed in a sitting or standing position. The assessments will be repeated three times after adequate rest. The maximum number of attempts permitted will be eight. Following the fulfilment of acceptability and repeatability criteria, the two best curves and two best tests will be selected. The average values of the FVC and FEV_1_ will be recorded (Hall & Stanojevic 2019).

### Six-minute walk test

The 6MWT is a valid, responsive and reliable outcome measure frequently used in cardiac and pulmonary rehabilitation. It is often used to determine functional exercise capacity in patients undergoing cardiorespiratory rehabilitation. In a review of 167 articles between 1948 and April 2011 through Ovid MEDLINE, SPORTS, EMBASE, CINAHL, Cochrane reviews and Cochrane clinical trial, it revealed a responsiveness of the 6MWT in cardiac rehabilitation with an estimated mean difference of 60.43 m (95%, confidence interval 54.57 m – 66.30 m; *p* < 0.001) and moderate evidence of repeatability (Bellet, Adams & Morris 2012). A study by Brown and Nathan (2018) revealed a strong correlation between clinical outcomes and 6MWT distance in patients with chronic respiratory diseases.

The 6MWT will be performed according to the standardised procedure, and the first author (PI) who will be blinded to group allocation will supervise the process. The participants will be asked to walk at their maximum pace along a 30 m-long straight road. They will not be given any encouragement; the patient’s symptoms may limit the test. Therefore, if there are any signs of significant distress, such as dyspnoea, dizziness, angina or skeletal muscle pain, the participant will be asked to stop and sit down. The participants will only be allowed to continue upon alleviation of the discomfort. The total distance the participants cover will be recorded in meters (Du Bois et al. 2011). The procedure will be repeated at a 2-week interval for the duration of our study.

### Medical Research Council dyspnoea scale

The MRC breathlessness scale quantifies the disability associated with breathlessness by identifying if breathlessness occurs when it should not (Grades 1 and 2) or by quantifying the associated exercise limitation (Grades 3–5) (Stenton 2008). Mahler and Wells (1988) reported a 98% agreement between observers recording MRC breathlessness scores. In a statement by the ATS, the score correlates well with the results of other breathlessness scales, lung function measurements and direct measures of disability such as walking distance (stats.oarc.ucla.edu). The MRC breathlessness scale is commonly used to describe patient cohorts and stratify them for pulmonary rehabilitation interventions, predict survival and use as complementary to FEV_1_ in describing disability in those with COPD and other forms of respiratory impairments (Bestall et al. 1999; Nishimura et al. 2002; Wedzicha et al. 1998).

The instrument has five statements that describe the range of respiratory disability. Depending on the literacy of the participants, the instrument can either be self or interviewer administered; RA2 will explain the content of the instrument to the participants prior to its administration. Participants will be encouraged to clarify issues related to the instruments before submitting their responses. The instrument will be administered at our study site at 2 week intervals during the study period. They will be comfortably seated on a chair without wheels, and the research instrument and writing materials placed on a desk. The RA2 will carefully introduce the research instrument to the participants, explain the domains and indicate the options stated on the instruments. Respondents will be informed to seek clarification on the instruments whenever required from either the PI or RA2, both blinded to group allocation.

### Pulmonary functional status and dyspnea questionnaire

The pulmonary functional status and dyspnea questionnaire (PFSDQ) is a self-administered questionnaire with 164 items designed to assess the level of dyspnoea and activities in respiratory conditions (Meek & Lareau 2003). The modified version of the PFSDQ-M is a shorter version of the instrument consisting of 40 items that evaluate dyspnoea, fatigue and levels of activity (Lareau, Meek & Roos 1998).

The PFSDQ and PFSDQ-M questionnaires evaluate dyspnoea independent of activities with five items that evaluate dyspnoea. Pulmonary functional status and dyspnea questionnaire measures dyspnoea associated with 79 activities. Pulmonary functional status and dyspnea questionnaire-modified measures dyspnoea associated with 10 activities (Meek & Lareau 2003).

The PFSDQ has good test–retest reliability, r = 0.94 on the dyspnoea scale and internal consistency of α = 0.88 to 0.94, while PFSDQ-M has a test–retest reliability on the dyspnoea scale of r = 0.83 and internal consistency of α = 0.94 (Lareau et al. 1998). The PFSDQ has been shown to be responsive over time, while the PFSDQ-M has been shown to be more responsive to change following pulmonary rehabilitation (Lareau et al. 1999).

The PFSDQ-M evaluates the effect of dyspnoea, fatigue and changes experienced in activities of daily living (ADL), the dyspnoea and fatigue domain of the instrument has five general items and 10 specific items, while the change experienced by patients has 10 specific items (Lareau et al. 1998). Participants are expected to assign a score according to their experience using a scale of 0–10 on the specific item scale for dyspnoea and fatigue with 0 score indicating no interference; 1–3 indicates mild interference; 4–6, moderate interference; 7–9 severe interference and 10 as extreme interference (Kovelis et al. 2008). For the third domain, participants are also required to quantify on a scale of 0–10 with 0 indicating as active always; 1–3 indicates slight change; 4–6 indicates moderate change; 7–9 indicates extreme change, while 10 indicates patient can no longer perform the activity (Kovelis et al. 2008). A partial score of 0 to 100 will be calculated for each domain while the overall score will be 0–300, with a higher value indicating the severity of activity limitation. The instrument will be administered every 2 weeks during our study period. Participants will be comfortably seated on a chair without wheels and the research instrument and writing materials will be placed on a desk. The RA2 will carefully introduce the research instrument to the participants, explain the domains and indicate the options stated on the instruments. Respondents will be informed to seek clarification on the instruments whenever required from either the PI or RA2, blinded to group allocation.

### Data analysis

The Statistical Package for Social Sciences (SPSS Inc, Chicago, Illinois, United Sates) version 26.0 for windows package programme will be used to analyse data. Descriptive statistics of mean, standard deviation, frequencies and percentages will be used to summarise the results. Bar charts, pie charts and histograms will be utilised for pictorial illustration. A multivariate analysis of variance (MANOVA) will be used to compare the outcome pulmonary function variables (FEV_1_, FVC, FEV_1_/FVC, PEFR) and QoL (6MWT, MRC dyspnoea scale score and PFSDQ-M score) among groups, while the Bonforreni’s *t*-test will be utilised for post hoc analysis to detect where significant changes occurred in the outcome variables. Dependent t-tests will be used to compare the pre- and post-test results within variables while independent t-tests will be used to compare the outcome variable among the study and control groups. The level of significance will be set at *p* ≤ 0.05.

The study does not potentially involve risk or harm, as all interventions can only be performed within the limit of the patient’s tolerance. However, participants may experience mild discomfort because of the exercise, including temporary muscle soreness, increased heart rate, blood pressure, sweating and dizziness. All necessary care will be in place to prevent the occurrence of any adverse event. However, in case of a report of serious adverse events (e.g. comorbidities, injuries, persistent excruciating pain, dizzy spells, headache, etc.) after the intervention or at any point during the trial, we would consider unblinding the participant to the intervention for their safety. Additionally, the participants will be instructed to report adverse events to the first author PI or the physiotherapist supervising their group. To ensure adequate supervision, participants per group to be attended in a day will be limited to a maximum of three. Arrangements will be made with the Accident and Emergency units of the research site to provide a standby medical team. The University of KwaZulu-Natal insurance scheme on clinical trials has fully covered participants in this type of study.

To promote participant retention, prevent loss to follow-up, compensate for time, inconveniences and expenses, participants with financial challenges accessing our study site will be granted a financial remuneration following international ethical guidelines and the South African Department of Health framework guidelines. The payment will be commensurate with the unskilled labour rate recommended by global ethics and South African guidelines (Ndebele et al. 2014). As our study is to be performed in Nigeria, Nigerian regulations regarding patient compensation will be considered. In Nigeria, unlike in South Africa, there is no minimal benchmark for payment of research participants; however, the *Labour Act* stipulates a minimum wage of 30 000.00 naira (Nigerian naira) per month to all unskilled workers, which is equivalent to $85.00 and amounts to $4.50/day. This amount was considered fair and reasonable by the Research Ethical Committee (REC) of UKZN, and the hospital facilities used as our study setting in Nigeria.

## Discussion

Despite a good number of randomised control trials on the effectiveness of incentive spirometry in the management of respiratory impairments, there is limited evidence that identifies the suitability of incentive spirometry (IIS or EIS) exercise for obstructive, restrictive and mixed respiratory impairments, respectively.

The outcome of our study may become an objective clinical tool in the rehabilitation of respiratory impairments, which will serve as an evidence-based approach in the physiotherapy management of the various classes of respiratory impairments. Furthermore, the outcome may create avenues for future research in this area and enhance the training of clinicians who have a special interest in cardiopulmonary physiotherapy.

Finally, it is expected that our findings may be recommended in clinical guidelines for managing respiratory impairments and will support the cost-benefit of managing respiratory impairments in Nigeria and other low-income countries.

## Access to protocol

https://pactr.samrc.ac.za/TrialDisplay.aspx?TrialID=11069. The protocol was registered on 17 May 2020 with identifier number PACTR202005904039357 and the trial organisation is PACTR.
